# Effects of w/b Ratio on Sodium Sulfate Crystallization Damage and Degradation Mechanisms in Semi-Immersed Alkali-Activated Slag Mortar

**DOI:** 10.3390/ma18132988

**Published:** 2025-06-24

**Authors:** Zhenwei Zhou, Yuetao Qiu, Peng Liu, Jianxiong Ye, Kunpeng Yin, Linwen Yu, Changhui Yang

**Affiliations:** 1 China Communications Construction Company Ltd., Beijing 100088, China; 18677177197@163.com; 2 Chongqing Jianyan Kezhijie New Material Co., Ltd., Chongqing 402761, China; jkarus@lets.com (Y.Q.); 13002263912@163.com (P.L.); 3 College of Materials Science and Engineering, Chongqing University, Chongqing 400045, China; linwen.yu@cqu.edu.cn (L.Y.); ychh@cqu.edu.cn (C.Y.)

**Keywords:** alkali-activated slag, sulfate crystallization, semi-immersion, pore structure, ion migration, efflorescence

## Abstract

This study investigates the long-term durability and crystallization-induced degradation mechanisms of alkali-activated slag (AAS) mortars with varying water-to-binder ratios (w/b, 0.4, 0.45, 0.5) under semi-immersion in 5 wt.% sodium sulfate solution. Through 360 d of exposure, the evolution of physical–mechanical properties (mass change, open porosity, compressive/flexural strength) and ion migration patterns (SO_4_^2−^, Na^+^, Ca^2+^) were analyzed to unravel the interplay between pore structure, ion transport, and crystallization-induced deterioration. Results demonstrated that higher w/b ratios exacerbated surface crystallization and spalling due to accelerated ion transport and pore coarsening. Early-stage strength gains (up to 25.15% at 120–180 d) stemmed from pore refinement via sulfate deposition and continued slag hydration. However, prolonged exposure triggered microstructural degradation, with open porosity increasing by 58.9% and strength declining by 30.6% at 360 d for a w/b of 0.5 compared to a w/b of 0.4. This was driven by crystallization pressure and the decalcification of hydration products. Ion migration analysis revealed SO_4_^2−^ enrichment in evaporation area and outward Na^+^ diffusion, establishing supersaturation gradients that aligned with crystallization damage progression. These findings provide critical insights for optimizing AAS mortar formulations to mitigate sulfate crystallization risks in semi-immersed environments.

## 1. Introduction

Concrete infrastructures in sulfate-rich environments, such as coastal zones and saline soils, are prone to severe durability degradation due to sulfate attack [1,2,3,4]. The Ordinary Portland cement-based materials often suffer from chemical sulfate corrosion, leading to expansion and cracking via the formation of ettringite and gypsum [5,6,7], and also its own alkali–silica reaction will cause it to swell and crack [8]. In contrast, Alkali-Activated Slag Cement (AASC), a sustainable alternative synthesized from granulated blast furnace slag (GBFS) and alkali-activators, exhibits excellent resistance to chemical sulphate attack by avoiding such expansive products [9,10]. However, recent studies highlight a critical vulnerability of AASC under cyclic wet-dry conditions, where sodium sulfate crystallization induces physical damage through pore-wall pressure, even without chemical corrosion products [11]. This paradoxical combination of excellent chemical resistance and a high risk of crystalline damage poses significant challenges for AASC applications in semi-immersed environments like tidal zones.

Under semi-immersion, capillary transport and evaporation synergistically drive sulfate ion enrichment, accelerating supersaturation and surface crystallization [12]. The AASC inherent characteristics exacerbate this process: its unsaturated water absorption rate is 2–3 times higher than Ordinary Portland cement concrete (OPC), and its pore structure dominated by sub-20 nm capillaries, amplifies crystallization pressure due to the inverse relationship between pore size and crystallization stress [13,14]. Furthermore, the high alkali ion concentration (e.g., Na^+^) in AASC pore solutions promotes sodium sulfate supersaturation, increasing crystallization potential [15]. Under cyclic wet-dry conditions, capillary transport and evaporation synergistically concentrate dissolved ions at exposed surfaces, leading to supersaturation and crystallization of salts like Na_2_SO_4_. This process exerts physical stress on pore walls, causing spalling and strength loss [16,17]. Unlike chemical sulfate attack, efflorescence in AAMs is primarily a physical degradation mechanism, exacerbated by their fine capillary pores (<20 nm) and high alkali content. Despite these risks, systematic investigations into the interplay between w/b ratios, ion migration, and microstructural evolution under semi-immersion remain scarce. Existing studies primarily focus on full-immersion scenarios or dry-wet cycles, overlooking the unique ion transport dynamics in partially saturated systems.

Recent advances highlight material-specific strategies: calcined clay-geopolymer hybrids reduce Na^+^ mobility through ion-exchange capacity [18], while hybrid alkali-activated cements (HAC) blend slag with OPC to enhance crystallization resistance via pore refinement [19]. However, neither approach resolves the intrinsic trade-off between workability (governed by w/b) and crystallization durability in pure AAS systems—the core focus of this study.

Previous work by our group revealed severe surface spalling (15% mass loss) in AAS mortars after 450 d of semi-immersion in 5 wt.% Na_2_SO_4_ solution, far exceeding damage under full immersion [11,13]. This underscores the critical role of evaporation-driven ion enrichment in crystallization damage. However, the mechanism linking the w/b ratio, a key parameter controlling the pore structure and transport properties, to the kinetics of sulphate crystallization and mechanical degradation has yet to be deciphered. Higher w/b ratios typically increase porosity and ion mobility [20,21], potentially accelerating sulfate accumulation and damage progression. Conversely, optimized ratios might mitigate these effects by refining pore networks. Resolving this trade-off is essential for designing durable AASC structures in sulfate-exposed environments.

This study addresses these gaps by evaluating AAS mortars with w/b ratios of 0.4, 0.45, and 0.5 during 360 d semi-immersion in sodium sulfate. Mechanical properties (compressive/flexural strength), pore structure evolution (open porosity, water absorption), and ion distribution (SO_4_^2−^, Na^+^, Ca^2+^) were analyzed to unravel crystallization damage mechanisms. Key objectives include: (1) quantifying the impact of w/b ratios on surface crystallization and spalling; (2) correlating pore structure changes with strength degradation; and (3) mapping ion migration patterns to establish supersaturation gradients driving crystallization. The findings aim to provide actionable insights for optimizing AASC formulations, balancing workability and durability in semi-immersed applications.

## 2. Experimental

### 2.1. Materials

The granulated blast furnace slag (GBFS) was produced from Chongqing Iron and Steel Group (Chongqing, China), with a specific surface area of 398.7 m^2^/kg by Blain method, and the main compositions measured by X-ray Fluorescence Spectrometer (XRF) are shown in Table 1. The fine aggregate was Yueyang river sand with a fineness modulus of 2.6, apparent density of 2.53 g/cm^3^, and bulk density of 1.79 g/cm^3^, according to the Chinese standard GB/T-14684-2022 [22]. Sodium sulphate was anhydrous sodium sulphate produced by Chengdu Kelong Chemicals Co. (Chengdu, China) with a content of sodium sulphate of more than 99%.

### 2.2. Preparation of AAS Mortars

The AAS mortars with water-to-binder ratios (w/b) of 0.4, 0.45, and 0.5 were prepared using a constant alkali content of 4% Na_2_O and a binder-to-sand ratio of 1:3. The alkali activator solution (SiO_2_:Na_2_O molar ratio = 1.5) was prepared by dissolving sodium hydroxide pellets in deionized water, followed by mixing with sodium silicate solution.

The slag and sand were dry-mixed for 2 min in a planetary mixer. The alkali activator was added gradually, followed by 3 min of mixing. The mixtures were cast into 40 × 40 × 160 mm^3^ steel molds within 5 min of mixing completion, vibrated with cement mortar vibration compaction equipment to remove entrapped air, and covered with plastic film. Specimens were demolded after 24 h and cured at 20 ± 2 °C under >95% relative humidity (RH) for 28 d.

After curing, specimens were vertically semi-immersed to a depth of 4 cm in 5 wt.% Na_2_SO_4_ solution maintained at 20 ± 2 °C. The exposure test was conducted in a controlled environment (20 ± 2 °C, 60 ± 5% RH) to simulate tidal zone conditions. The solution was replenished every 60 d to maintain consistent ion concentration. The samples were labeled as AAS-0.4, AAS-0.45, and AAS-0.5 based on their w/b ratios. Detailed mix proportions are summarized in Table 2.

### 2.3. Experiment of the Semi-Immersion Form of AAS Sodium Sulphate Erosion

#### 2.3.1. Surface Crystallization and Mass Change

During the semi-immersion in sodium sulphate, crystalline features on the mortar surface were documented via digital imaging every 60 d. The mass of the mortar was determined using an electronic balance (RC-FA-1262SEM, Shanghai Sunyu Hengping Scientific Instrument Co., Shanghai, China, ±0.1 mg) after removing the crystalline material from the surface of the specimen after the photographs were taken, and the rate of change in the mass of the mortar during the erosion process was calculated according to Equation (1).

(1) $$M_{t}=\frac{m_{t}-m_{0}}{m_{0}}\;\times \;100\%$$

where: M_t_ was the mass change ratio, m_t_ referred to the mass of the AAS mortar after semi-immersion in a specific age, and m_0_ represented the initial mass of mortar before erosion.

#### 2.3.2. Mechanical Property

The flexural and compressive tests were carried out using DYE-10A Flexural Testing Machine for Mortar and NYL-600 Pressure Testing Machine (Beijing North Jianyi Technology Co., Beijing, China). The loading rates of flexural strength and compressive strength were 50 N/s and 2.4 kN/s according to the Chinese standard GB/T17671-2021 [24], and the test results were the average values of three specimens and six specimens, respectively.

#### 2.3.3. Pore Structure Analysis

The open porosity of the mortar was measured following the standard ASTM C642 [25,26]. Specimens were saturated in water for 72 h, surface-dried with a towel, and weighed in saturated surface-dry (m_s_) and submerged (m_w_) states. After oven-drying at 40 °C to constant weight, the dry mass (m_d_) was recorded. Total open porosity (φ_t_) was calculated as:

$$\varphi_{t}=\frac{m_{s}-m_{d}}{m_{s}-m_{w}}$$

To determine the water absorption coefficient (S), s the specimens were dried to constant weight at 40 °C, sealed on four sides with aluminum foil, and partially immersed in water (3–5 mm depth). Mass gain was monitored every 15 min, and S was derived by fitting the data to:

$$I=S\sqrt{t}$$

where I was the cumulative water absorption per unit area, and t was the absorption time.

### 2.4. Erosion Resistance Mechanism of AAS

**Figure 1:**
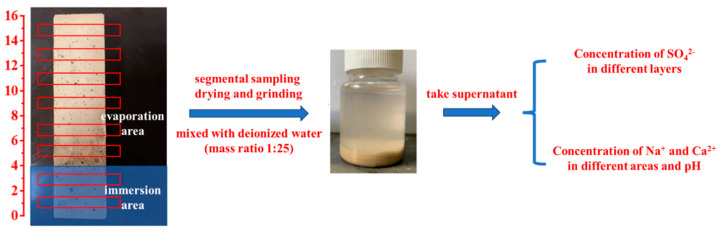
SO_4_^2−^, Na^+^, Ca^2+^ ion concentrations and pH measurement.

To analyze ion migration and pH evolution during erosion, SO_4_^2−^, Na^+^, and Ca^2+^ concentrations in leachates were quantified using a UV spectrophotometer (UV-2600, Shimadzu, Kyoto, Japan), ICP-OES (ICAP6300, Thermo Fisher Scientific, Shanghai, China), and a pH meter (TP310, Beijing Times New Dimension Measurement and Control Equipment Co., Beijing, China). Mortar specimens at designated ages (0–360 d) were retrieved, and surface crystallization products were mechanically removed. Before testing, seal all four sides of the mortar with aluminum foil tape and cover the top with a plastic sheet to prevent water evaporation. Then the specimens (40 × 40 × 160 mm^3^) were vertically semi-immersed to 4 cm depth in 5 wt.% Na_2_SO_4_ solution. As illustrated in Figure 1, the immersion and evaporation areas were separately sectioned using a concrete powder mill. Powder samples were collected at 1–15 cm intervals from the specimen base, sieved (160 mesh), and dried to constant weight. The sieved powder was mixed with deionized water (1:25 mass ratio), sealed for 24 h, and filtered to obtain leachates for testing.

## 3. Results and Discussion

### 3.1. Macroscopic Performance Evolution Under Sulfate Attack

This section systematically evaluates the macroscopic degradation of AAS mortars under semi-immersion in sodium sulfate, focusing on surface deterioration, mass dynamics, mechanical degradation, and pore structure evolution.

#### 3.1.1. Surface Crystallization and Morphological Deterioration

**Figure 2:**
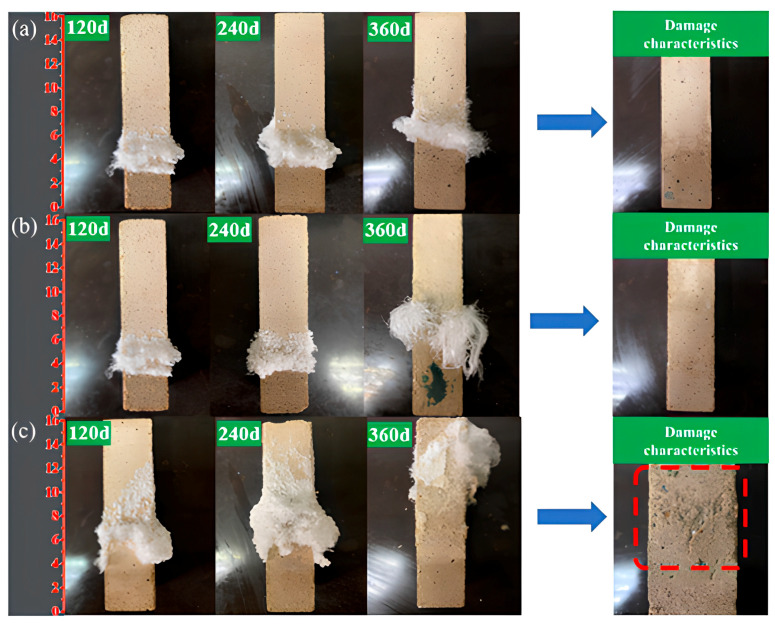
Apparent crystalline behavior and damage characteristics of AAS mortars with different w/b ratios at immersion in 5 wt.% Na_2_SO_4_ solution ((**a**): AAS-0.4, (**b**): AAS-0.45, (**c**): AAS-0.5).

The surface crystallization behavior of AAS mortars under semi-immersion in sodium sulfate solution exhibited significant dependency on the water-to-binder (w/b) ratio. As illustrated in Figure 2, sodium sulfate crystals progressively accumulated on the evaporation area of the mortar surface, with crystallization intensity escalating as the w/b ratio increased. At 120 d of exposure, the AAS-0.5 specimen (w/b = 0.5) displayed extensive crystal coverage (about 35% of the evaporation area), whereas AAS-0.4 (w/b = 0.4) and AAS-0.45 (w/b = 0.45) exhibited smaller crystallization (about 20% coverage). The AAS-0.5 surface was almost entirely encrusted with crystalline deposits at 360 d (Figure 2c), accompanied by severe spalling in the evaporation area, while AAS-0.4 and AAS-0.45 retained structural integrity despite localized crystal formation.

**Figure 3:**
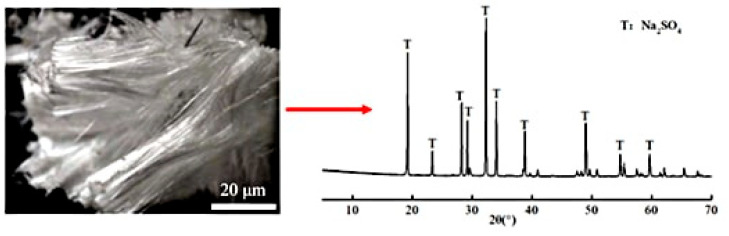
Crystalline products on the mortar surface and the XRD patterns.

XRD analysis (Figure 3) confirmed the crystalline deposits as anhydrous sodium sulfate (Na_2_SO_4_), formed through supersaturation driven by capillary-evaporation synergy. The sodium sulfate solution infiltrated the mortar matrix via capillary action, with subsequent evaporation concentrating ions at the air-exposed surface [11,27]. Higher w/b ratios amplified this process due to accelerated ion mobility and coarsened pore structures, which facilitated rapid sulfate transport and prolonged supersaturation.

The spalling severity in high w/b specimens (e.g., AAS-0.5) arose from crystallization pressure exerted within pores. As Na_2_SO_4_ crystals nucleated and grew, the resultant stress exceeded the strength of the mortar matrix, particularly in the evaporation area where porosity and water absorption were elevated. Conversely, lower w/b ratios (0.4 and 0.45) mitigated damage through refined pore networks, which restricted ion ingress and distributed crystallization stresses more uniformly.

These observations are in agreement with prior studies [9,28], confirming that AAS mortars resist chemical sulfate attack (e.g., ettringite formation) but remain vulnerable to physical crystallization damage under semi-immersion. The interplay between w/b ratio, pore structure, and ion transport dynamics underscores the critical role of formulation optimization in balancing durability and crystallization resistance.

#### 3.1.2. Mass Change and Spalling Behavior

**Figure 4:**
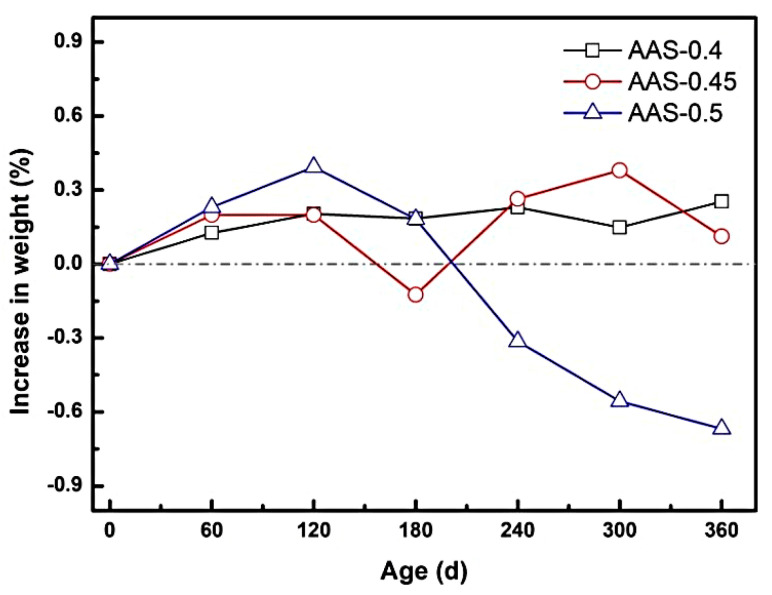
Mass change of AAS mortar with different w/b ratios during the immersion process.

The mass evolution of AAS mortars during semi-immersion in sodium sulfate solution revealed distinct phases of sulfate accumulation and crystallization-induced spalling, strongly influenced by the water-to-binder (w/b) ratio. As depicted in Figure 4, mortars with lower w/b ratios (0.4 and 0.45) exhibited a gradual mass gain during the erosion process, where the increases at 360 d were 0.25% and 0.13%, respectively. This trend reflects the progressive infiltration and deposition of sodium sulfate within capillary pores, enhanced by the continuous hydration of unreacted slag particles [29].

In contrast, the AAS-0.5 specimen (w/b = 0.5) displayed a biphasic mass response: an initial rapid gain of 0.39% at 120 d, followed by a sharp decline to a net loss of 0.67% by 360 d. The pore structure was decisive for this process, as evidenced by the water absorption coefficient of AAS-0.5 much higher than that of AAS-0.4 and AAS-0.45, indicating significantly enhanced pore connectivity. The early mass increase stemmed from accelerated sulfate ingress due to the coarser pore structure and elevated water absorption coefficient, which facilitated capillary-driven ion transport. This high connectivity promoted rapid SO_4_^2−^/Na^+^ migration to the evaporation area, concentrating ions for crystallization. However, prolonged exposure led to supersaturation of Na_2_SO_4_ in the evaporation area, triggering extensive crystallization. The resultant crystallization pressure, amplified by the inverse relationship between pore size and stress intensity, exceeded the mortar strength, with the interconnected pore network further aggravating matrix exfoliation after crystal formation, causing surface spalling and subsequent mass loss [30,31].

The change in mass of AAS mortar reflects its spalling condition. The spalling severity correlated directly with w/b ratio. For w/b = 0.5, macroscopic flaking of the surface layer in the evaporation area (Figure 2c), while lower w/b ratios (0.4 and 0.45) showed no visible spalling. This divergence arises from the interplay between pore refinement and transport kinetics: lower w/b mortars restricted sulfate accumulation and distributed crystallization stresses more uniformly, delaying damage initiation.

#### 3.1.3. Mechanical Degradation: Compressive and Flexural Strength

The mechanical performance of AAS mortars under semi-immersion in sodium sulfate solution exhibited dual-phase behavior: initial strength enhancement followed by progressive degradation, with the severity of deterioration governed by the water-to-binder (w/b) ratio.

**Figure 5:**
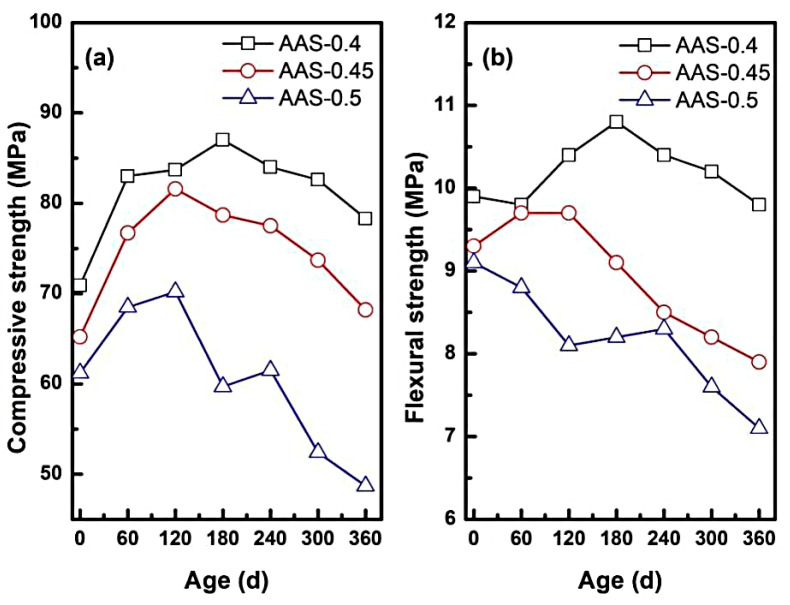
Mechanical properties of AAS mortar with different w/b ratios during erosion ((**a**): compressive strength, (**b**): flexural strength).

As shown in Figure 5a, compressive strength initially increased for all w/b ratios, peaking at 180 d for AAS-0.4 (+22.71%), 120 d for AAS-0.45 (+25.15%), and 120 d for AAS-0.5 (+14.71%). Early strength gains align with slag hydration kinetics [32] and sulphate-induced [9,33] pore refinement. In this process, residual slag reaction and sodium sulphate deposition work together to enhance matrix density. (1) Pore refinement: sulfate ions (SO_4_^2−^) migrating into capillary pores precipitated as Na_2_SO_4_ crystals, reducing pore volume and enhancing matrix compactness. (2) Continued slag hydration [29,32]: residual unhydrated slag particles reacted with alkaline activators, forming additional C-(N)-A-S-H gel that strengthened the binder network. However, prolonged exposure triggered strength loss, with compressive strength declining by 10% (w/b = 0.4), 16.42% (w/b = 0.45), and 30.63% (w/b = 0.5) at 360 d (Figure 5a). This degradation might be correlated with two mechanisms: (1) crystallization pressure [34]: supersaturated Na_2_SO_4_ in coarser pores (w/b = 0.5) generated internal stresses exceeding the tensile strength of the matrix, inducing microcracks and porosity expansion. (2) Decalcification of hydration products [35]: leaching of Ca^2+^ from C-(N)-A-S-H gel weakened the gel structure, particularly in high w/b mortars, where transport properties accelerated ion exchange.

Flexural strength followed similar biphasic trends for w/b = 0.4 and 0.45, peaking at 120 d and 180 d before declining (Figure 5b). In contrast, w/b = 0.5 exhibited monotonic flexural strength reduction (26.38% at 360 d). The reason for this discrepancy could be: (1) silica-rich gel bridging [11]: decalcification of C-(N)-A-S-H gel produced silica-rich phases that temporarily bridged microcracks, mitigating early flexural loss in lower w/b mortars. (2) pore coarsening: higher w/b ratios (0.5) exacerbated pore coarsening, diminishing the reinforcing effect of silica-rich gel and amplifying stress concentrations under bending loads [36].

These results demonstrated that the refined pore network with low w/b (0.4–0.45) slowed down the sulfate intrusion and crystalline stress distribution, and retarded the strength loss. However, the coarsened pores with high w/b (0.5) accelerated the ion migration, which exacerbated the supersaturation gradient and microstructural damage.

#### 3.1.4. Pore Structure Evolution and Water Absorption Dynamics

**Figure 6:**
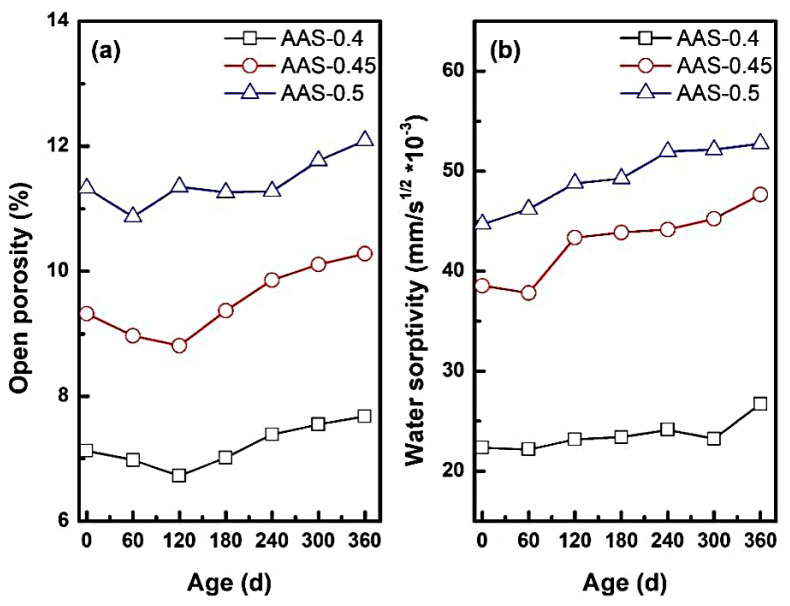
Open porosity and water absorption rate coefficient of AAS mortar with different w/b ratio ((**a**): open porosity, (**b**): water absorption coefficient).

The open porosity and water absorption coefficient could reflect the change of internal pore structure of AAS mortar. It also determined the transport rate of sodium sulfate solution inside the AAS mortar, reflecting the internal microstructural changes of the mortar during the erosion process. Figure 6 presents the variation of open porosity and water absorption coefficient of AAS mortar with different w/b ratios during the erosion process.

As shown in Figure 6a, open porosity exhibited a biphasic trend during erosion: initial reduction followed by significant expansion. For example, AAS-0.4 and AAS-0.45 reached a porosity minimum at 180 d, attributed to pore refinement via sulfate deposition and continued slag hydration [37,38]. Sodium sulfate crystals filled capillary pores, reducing connectivity and enhancing matrix compactness. However, prolonged exposure reversed this trend, with open porosity increasing by 6.6% for w/b = 0.5 compared to its initial state. This degradation stemmed from crystallization pressure within coarser pores (dominant in high w/b mortars), which generated microcracks and expanded pore networks.

The w/b ratio critically modulated porosity dynamics. Mortars with w/b = 0.5 exhibited the highest open porosity, nearly 1.6 times that of w/b = 0.4. This disparity arose from the coarser pore structure in high w/b mortars, which accelerated sulfate ingress and localized stress concentrations during crystallization.

Water absorption coefficients (Figure 6b) increased progressively with erosion time, reflecting pore structure degradation. For w/b = 0.5, the absorption coefficient surged by 23.52% (from 44.72 to 52.77 g/m^2^·s^−1^) at 360 d. This might be attributed to: (1) pore connectivity: higher w/b ratios amplified pore interconnectivity, creating preferential pathways for water and ion transport. (2) Hydration of anhydrous Na_2_SO_4_: during drying cycles, anhydrous Na_2_SO_4_ in pores absorbed moisture to form hydrated phases (e.g., Na_2_SO_4_·10H_2_O), further increasing water uptake.

These findings underscore the critical role of w/b ratio in balancing pore structure refinement against crystallization risks, providing actionable guidelines for optimizing AAS formulations in sulfate-rich environments.

### 3.2. Mechanistic Insights into Sulfate-Induced Degradation

#### 3.2.1. Analysis of SO_4_^2−^ Ion Migration Behavior

**Figure 7:**
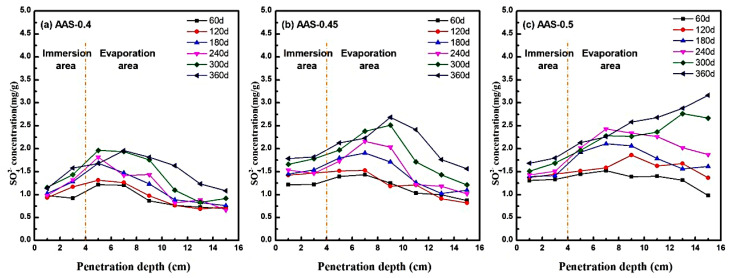
SO_4_^2−^ distribution characteristics of AAS mortars with different w/b ratios at different erosion ages ((**a**): AAS-0.4, (**b**): AAS-0.45, (**c**): AAS-0.5).

The migration and enrichment of SO_4_^2−^ ions within AAS mortars under semi-immersion were systematically analyzed by measuring spatial ion concentrations at incremental depths (1–15 cm from the base) using UV spectrophotometry. Figure 7 illustrates the SO_4_^2−^ distribution profiles across immersion and evaporation areas for mortars with varying w/b ratios (0.4, 0.45, 0.5) at 360 d of exposure.

In low w/b mortars (e.g., AAS-0.4), SO_4_^2−^ exhibited limited transport capacity, peaking at 2.0 mg/g at 7 cm from the base (evaporation area) before declining sharply to 1.0–1.25 mg/g at greater distances (Figure 7a). This localized enrichment aligns with capillary-evaporation dynamics: finer pore networks restricted ion mobility, causing sulfate accumulation in the mid-evaporation region. Conversely, higher w/b ratios amplified transport kinetics. For AAS-0.5 (Figure 7c), SO_4_^2−^ concentrations increased monotonically with distance, reaching 3.2 mg/g at the top of the evaporation area. The coarser pore structure facilitated rapid capillary rise and prolonged ion retention, enabling sulfate to permeate the entire evaporation area.

The biphasic concentration trends, which rise in the immersion area and peak in the mid-evaporation regions, reflect the balance between the ingress driven by capillaries and the supersaturation driven by evaporation [39,40]. This mechanism is consistent with Fickian diffusion models [41], where ion flux (J) is proportional to the concentration gradient (∇C) and diffusivity (D): J=−D∇C. Higher w/b ratios elevated D due to increased pore connectivity, accelerating sulfate ingress. Concurrently, evaporation at the surface established a supersaturation gradient, promoting Na_2_SO_4_ crystallization in regions with sufficient ion accumulation.

Notably, the spatial mismatch between SO_4_^2−^ enrichment and crystallization damage highlight the role of pore size distribution. In AAS-0.4, sulfate is concentrated in sub-20 nm pores, where crystallization stress inversely scales with pore radius. Despite lower total sulfate content, localized stress exceeded matrix strength in coarser pores, initiating microcracks. In contrast, AAS-0.5 broader pore distribution dispersed stress but allowed deeper sulfate penetration, exacerbating surface spalling.

These findings underscore the critical trade-off between transport kinetics and crystallization resistance. Lower w/b ratios mitigate damage by refining pore networks, while higher ratios amplify sulfate ingress and supersaturation risks.

#### 3.2.2. Na^+^ Ion Redistribution and Crystallization Influence

The redistribution of Na^+^ ions within AAS mortars under semi-immersion played a pivotal role in governing sodium sulfate crystallization dynamics, particularly due to the inherently high Na^+^ content in alkali-activated systems.

**Figure 8:**
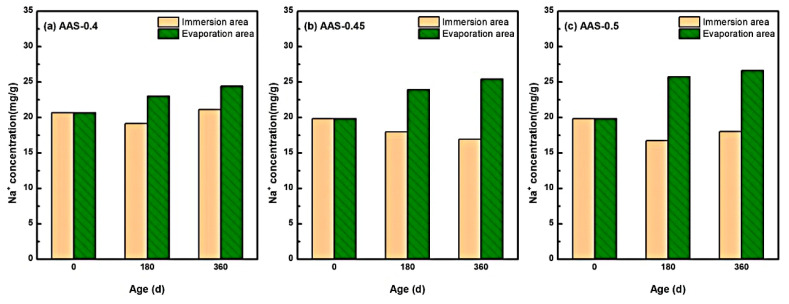
Characteristics of Na^+^ distribution of AAS mortars with different w/b ratios at different erosion ages ((**a**): AAS-0.4, (**b**): AAS-0.45, (**c**): AAS-0.5).

Figure 8 delineates the spatial-temporal evolution of Na^+^ concentrations across immersion and evaporation areas for mortars with varying w/b ratios (0.4, 0.45, 0.5). Notably, Na^+^ exhibited a bidirectional migration pattern: outward diffusion from the immersion area (saturated region) and upward capillary-driven transport toward the evaporation area (unsaturated region). For instance, in AAS-0.5 (w/b = 0.5), Na^+^ concentration in the immersion area decreased by 9.10% over 360 d, while the evaporation area exhibited a 34.26% increase. In contrast, lower w/b ratios (0.4 and 0.45) displayed attenuated Na^+^ migration, with evaporation area concentrations rising by only 19.03% and 28.16%, respectively.

This behavior stems from the interplay of three mechanisms: (1) Concentration-driven diffusion [42]: the initial Na^+^ concentration in AAS pore solutions exceeded that of the external sodium sulfate solution (5 wt.%), establishing a concentration gradient that drove outward Na^+^ diffusion from the immersion area. (2) Capillary-evaporation synergy: in the evaporation area, continuous water loss elevated ionic concentrations, creating localized supersaturation [43]. Concurrently, capillary rise transported Na^+^ and SO_4_^2−^ from the immersion area, further amplifying ion accumulation. (3) Pore structure modulation: Higher w/b ratios (e.g., 0.5) exacerbated pore coarsening, accelerating ion mobility and prolonging Na^+^ retention in the evaporation area.

These findings align with studies by, which emphasize that Na^+^ migration governs crystallization kinetics in alkali-activated systems. However, this work uniquely quantifies the role of w/b ratios in modulating Na^+^ transport pathways and crystallization regimes, providing a mechanistic basis for optimizing pore structures to mitigate damage.

#### 3.2.3. pH Evolution and Alkalinity Loss

**Figure 9:**
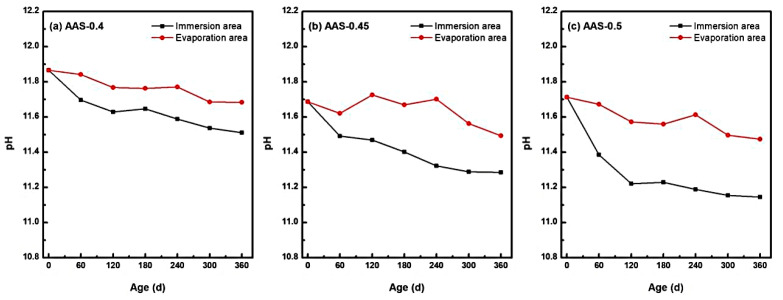
pH changes of AAS mortars with different w/b ratios at different erosion ages ((**a**): AAS-0.4, (**b**): AAS-0.45, (**c**): AAS-0.5).

The long-term stability of hydration products in alkali-activated slag (AAS) mortars is intrinsically linked to their high-alkalinity environment, which was critically influenced by semi-immersion in sodium sulfate solution [44]. As shown in Figure 9, pH values in both immersion and evaporation areas exhibited a progressive decline over 360 d of exposure, with distinct spatial and compositional dependencies tied to w/b ratios.

The immersion zone, characterized by continuous water saturation, experienced the most pronounced alkalinity loss. For AAS-0.5 (w/b = 0.5), pH decreased by 4.85% (from 11.712 to 11.144), while AAS-0.4 (w/b = 0.4) and AAS-0.45 (w/b = 0.45) showed reductions of 3.06% and 3.47%, respectively. This accelerated pH declined in high w/b mortars arose from enhanced ion mobility through coarser pore networks, facilitating outward leaching of hydroxyl ions (OH^−^) and alkali ions (Na^+^) into the external solution. The sustained ionic exchange destabilized hydration products, particularly C-(N)-A-S-H gel, promoting decalcification reactions and free Ca^2+^ release [5].

In contrast, the evaporation area exhibited milder pH reductions (e.g., 2.04% for AAS-0.5), attributed to limited water availability and localized ion enrichment. Evaporation-driven concentration effects partially counteracted OH^−^ leaching, yet residual moisture still enabled gradual alkalinity loss. Notably, the pH gradient between immersion and evaporation areas widened with higher w/b ratios, reflecting divergent transport kinetics.

The pronounced alkalinity reduction in immersion area underscores the vulnerability of AAS mortars to chemical destabilization under semi-immersion. Lower w/b ratios mitigated this degradation by refining pore structures, thereby preserving the high-pH environment essential for hydration product stability. These findings highlight the necessity of optimizing w/b ratios to balance ionic mobility and alkalinity retention in sulfate-rich environments.

#### 3.2.4. Decalcification and Free Ca^2+^ Release

**Figure 10:**
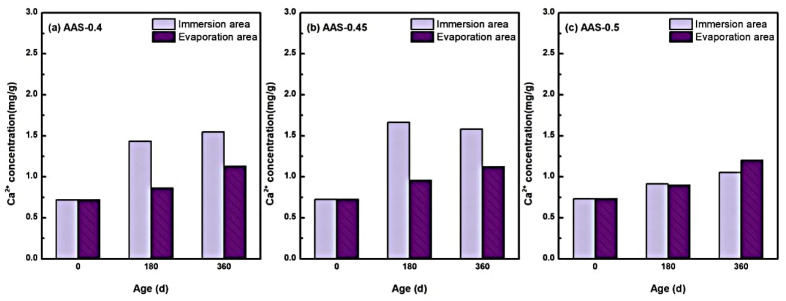
Variation of free Ca^2+^ concentration in the interior of AAS mortar with different w/b ratio ((**a**): AAS-0.4, (**b**): AAS-0.45, (**c**): AAS-0.5).

To further investigate the chemical stability of the hydration products during the erosion process, the changes in the internal free Ca^2+^ concentration of AAS mortars with different w/b ratios were analyzed during the immersion process, and the results are shown in Figure 10.

Free Ca^2+^ concentrations exhibited a marked increase across all w/b ratios during the 360 d erosion period, with pronounced spatial heterogeneity between immersion and evaporation areas. For instance, in AAS-0.4 (w/b = 0.4), Ca^2+^ concentration in the immersion area surged by 115.30% (from 0.719 to 1.548 mg/g), compared to a milder 57.02% increase (from 0.719 to 1.129 mg/g) in the evaporation zone. This disparity underscores the dominant role of continuous water saturation in the immersion area, which facilitated ionic dissolution and decalcification of hydration products, particularly C-(N)-A-S-H gel [45].

The decalcification process intensified with higher w/b ratios but exhibited nonlinear behavior. AAS-0.45 (w/b = 0.45) showed the most significant Ca^2+^ accumulation in the immersion area, reflecting enhanced ion mobility through moderately coarsened pores. However, for AAS-0.5 (w/b = 0.5), despite severe pH reduction in the immersion area, Ca^2+^ concentration increased only to 1.112 mg/g, which lower than expected. This anomaly arose from the interplay between accelerated decalcification and outward Ca^2+^ migration [46]: coarser pore networks enabled rapid leaching of liberated Ca^2+^ ions into the external solution, reducing their retention within the mortar matrix.

In contrast to the immersion area, Ca^2+^ enrichment in the evaporation area remained limited (e.g., 1.129 mg/g for w/b = 0.4 at 360 d). Restricted water availability and localized sulfate crystallization suppressed sustained decalcification, while evaporation-driven ion concentration partially offset Ca^2+^ leaching. Notably, higher w/b ratios marginally amplified Ca^2+^ accumulation in the evaporation area (e.g., 1.233 mg/g for w/b = 0.5), consistent with broader ion transport pathways.

These findings reveal a dual role of w/b ratios in governing decalcification: (1) Lower ratios (0.4–0.45) restricted ion mobility, localizing Ca^2+^ release within the immersion area and exacerbating microstructural damage [47,48]. (2) Higher ratios (0.5) accelerated Ca^2+^ leaching, depleting internal concentrations despite intensified decalcification [49]. This trade-off underscores the importance of optimizing w/b ratios to balance pore structure refinement and ion retention, thereby mitigating long-term chemical degradation in sulfate-rich environments.

## 4. Discussion

The key degradation metrics across w/b ratios at 360 d are consolidated in Table 3 for direct comparison.

The degradation of AAS mortars in a semi-immersed state could be attributed to two synergistic mechanisms: physical crystallization pressure and chemical decalcification. Higher w/b ratios (0.5) accelerated both processes due to coarsened pores, increasing ion mobility by 58.03% and enabling deeper sulphate penetration. This is consistent with the findings of references [50,51], which noted that capillary-driven ion enrichment in coarser pores exacerbates efflorescence.

Notably, the biphasic strength evolution (an initial gain followed by a loss) contrasted with OPC systems, in which sulfate attack usually caused a steady deterioration via ettringite expansion [52,53,54]. In the AAS, the initial strength increase was due to pore-filling crystallization and an ongoing slag reaction, which was consistent with the findings of Rashad et al. [55]. However, prolonged exposure caused strength loss of over 30% in high w/b mortars, which was greater than the losses reported for hybrid alkali-activated cements [56] or fly ash-slag blends [57].

The alkali activation fundamentally reorganized the structure of slag by breaking Ca–O–Si bonds and releasing Ca^2+^ and silicate monomers, which then formed a C-(N)-A-S-H gel [58,59]. At an optimal dosage of 4% Na_2_O, this yielded a gel with a Ca/Si ratio of approximately 1.2 and an Al/Si ratio of approximately 0.3, achieving a balance of strength and durability. However, increasing the alkali concentration over-dissolved the slag glassy phase, resulting in increased free Na^+^ and pore coarsening [60], which exacerbated sulfate crystallization. In contrast, clay-based geopolymers fractured Al–O–Si bonds under alkali attack to form nanoporous N-A-S-H gels that immobilized Na^+^ via ion-exchange sites [61]. This structural distinction explained why AAS was more vulnerable to crystallization despite similar alkali dosages. The severity of efflorescence in AAS was three times higher than in clay-based geopolymers [61], which was attributable to the higher mobility of Na^+^ in AAS. Optimizing w/b to 0.4–0.45 reduced spalling by refining pores, a strategy similar to those proposed for mitigating efflorescence in natural pozzolan-based AAMs.

## 5. Conclusions

The following conclusions were drawn from the investigation of AAS mortars under semi-immersion in sodium sulfate solution:

The higher water-cement ratio (0.5) significantly exacerbated surface crystallization and spalling, with almost 100% Na_2_SO_4_ coverage in the evaporation area after 360 d. This damage originated from accelerated ion transport and pore coarsening, with local crystallization stresses exceeding the tensile strength of the mortar. Lower water-cement ratios (0.4–0.45) can maintain structural integrity by refining the pore network and delaying stress accumulation.

Early strength gains (up to 25.15%) were driven by pore refinement and slag hydration, while prolonged exposure caused strength losses (up to 30.63%) due to microcracking from crystallization pressure and decalcification of hydration products. Flexural strength trends further highlighted the role of silica-rich gel bridging in mitigating damage at lower w/b ratios.

The SO_4_^2−^ ion enrichment in evaporation area and outward Na^+^ diffusion established supersaturation gradients, aligning with crystallization progression. Higher w/b ratios amplified sulfate penetration depth (3.2 mg/g at 15 cm for w/b = 0.5), while lower ratios restricted transport (2.0 mg/g at 7 cm for w/b = 0.4).

The alkalinity loss (pH reduction up to 4.85%) and free Ca^2+^ release (115.3% increase in immersion area) underscored the vulnerability of hydration products to decalcification, exacerbated by coarser pore structures in high w/b mortars.

This study demonstrated that optimizing the w/b ratio (0.4–0.45) effectively balances pore refinement, ion retention, and stress distribution, providing a viable strategy for improving the durability of AAS mortars in sulphate-rich semi-immersed environments. Although a w/b ratio of 0.4–0.45 minimizes the risk of sulfate crystallization, real-world applications require control of alkali leaching (with an evaporation area of Na^+^ < 30% of the baseline) and monitoring of carbonation (with a pH level of >11.5). ASTM C642 pore data capture transport-relevant capillaries, but not gel-scale effects.

## Figures and Tables

**Table 1:** Compositions of GBFS (wt%).

Compositions	SiO_2_	CaO	Al_2_O_3_	MnO	Na_2_O	MgO	Fe_2_O_3_	K_2_O	LOI
Content	27.91	43.91	13.44	0.39	0.35	7.53	0.60	0.37	0.61

**Table 2:** Summarizes mix proportions and curing parameters.

Parameter	AAS-0.4	AAS-0.45	AAS-0.5	Standard/Note
w/b ratio	0.40	0.45	0.50	
Na_2_O content	4%	4%	4%	Slag mass basis
Binder:sand ratio	1:3	1:3	1:3	By mass
Activator SiO_2_:Na_2_O	1.5	1.5	1.5	Molar ratio
Curing conditions	20 ± 2 °C, >95% RH for 28 d			GB/T 17671-2021 [23]

**Table 3:** Consolidates key performance metrics at 360 d.

Property	AAS-0.4	AAS-0.45	AAS-0.5	Test Method
Mass change (%)	+0.25	+0.13	−0.67	Equation (1)
Compressive. strength loss (%)	−10.0	−16.4	−30.6	GB/T 17671-2021
Flexural strength loss (%)	−0.10	−15.05	−26.38	
Open porosity (%)	7.72	10.24	12.08	ASTM C642
SO_4_^2−^ at 15 cm * (mg/g)	1.10	1.55	3.20	UV spectrophotometry
Na^+^ mobility index * (%)	+19.03	+28.16	+34.26	ICP-OES
Free Ca^2+ #^ (mg/g)	1.548	1.550	1.112	
pH value change ^#^ (%)	−3.06	−3.47	−4.85	pH meter

The evaporation area is indicated by * and the immersion area by ^#^.

## Data Availability

The data presented in this study are available on request from the corresponding author due to containing proprietary formulations protected by industrial partners.
